# Characterization of two HIV-1 infectors during initial antiretroviral treatment, and the emergence of phenotypic resistance in reverse transcriptase-associated mutation patterns

**DOI:** 10.1186/s12985-015-0417-y

**Published:** 2015-11-14

**Authors:** Wei Guo, Jingwan Han, Daomin Zhuang, Siyang Liu, Yongjian Liu, Lin Li, Hanping Li, Zuoyi Bao, Fujiang Wang, Jingyun Li

**Affiliations:** Department of AIDS Research, State Key Laboratory of Pathogen and Biosecurity, Beijing Institute of Microbiology and Epidemiology, 20 Dongda Street, Fengtai District, Beijing 100071 China; NO. 201 hospital of the People’s Liberation Army of China, Liaoyang, China

**Keywords:** HIV-1, Resistance mutations, Recombinant virus, Phenotypic resistance

## Abstract

**Background:**

Highly active antiretroviral therapy (HAART) is recommended to control the infection of HIV-1. HIV-1 drug resistance becomes an obstacle to HAART due to the accumulation of specific mutations in the RT coding region. The development of resistance mutations may be more complex than previously thought.

**Methods:**

We followed two HIV-1 infectors from a HIV-1 drug resistance surveillance cohort in Henan province and evaluated CD4+ T-cell number and viral load thereafter at ten time-periods and characterized their reverse transcriptase-associated mutation patterns at each time point. Then we constructed the recombinant virus strains with these mutation patterns to mimick the viruses and test the phenotypic resistance caused by the mutation patterns on TZM-b1 cells.

**Results:**

CD4+ T-cell number initially increased and then decreased rapidly, while viral load decreased and then dropped sharply during initial antiretroviral treatment. The number of mutations and the combination patterns of mutations increased over time. According to the phenotypic resistance performed by recombinant virus strains, Virus*T215Y/V179E/Y181C/H221Y* exhibited high levels of resistance to EFV (5.57-fold), and T215Y/V179E-containing virus increased 20.20-fold in AZT resistance (*p* < 0.01). VirusT215Y/V179E/Y181C increased markedly in EFV resistance (*p* < 0.01). The IC50 for Virus*T215Y/V179E/H221Y* was similar to that for Virus*T215Y/V179E/Y181C*. Virus*T215Y/K103N/Y181C/H221Y* induced a dramatic IC50 increase of all the four agents (Efavirenz EFV, Zidovudine AZT, Lamivudine 3TC, and Stavudine d4T) (*p* < 0.01). As for Virus*T215Y/K103N/Y181C*, only the IC50 of EFV was significantly increased. T215Y/K103N resulted in a 26.36-fold increase in EFV (*p* < 0.01). T215Y/K103N/H221Y significantly increased the resistance to AZT and 3TC. The IC50 of EFV with T215Y/V179E was lower than with T215Y/K103N (F = 93.10, *P* < 0.0001). With T215Y/V179E, Y181C significantly increase in EFV resistance, while the interaction between 181 and 221 in EFV was not statistically significant (F = 1.20, *P* = 0.3052). With T215Y/K103N, neither H221Y nor Y181C showed a significant increase in EFV resistance, but the interaction between 181 and 221 was statistically significant (F = 38.12, *P* = 0.0003).

**Conclusions:**

Data in this study suggests that pathways of viral evolution toward drug resistance appear to proceed through distinct steps and at different rates. Phenotypic resistance using recombinant virus strains with different combination of mutation patterns reveals that interactions among mutations may provide information on the impact of these mutations on drug resistance. All the result provides reference to optimize clinical treatment schedule.

## Background

Highly active antiretroviral therapy (HAART) consists of two nucleoside reverse transcriptase inhibitors (NRTIs) plus a non-nucleoside reverse transcriptase inhibitor (NNRTI) in China. It highly suppresses HIV-1 replication and reduces the morbidity and mortality associated with HIV-1 infection and AIDS [1, 2]. However, HIV-1 drug resistance is becoming an obstacle to effective long-term HAART due to the accumulation of specific mutations in the reverse transcriptase (RT) coding region [3]. More than 200 mutations are associated with antiretroviral resistance and HIV-1 drug resistance profiles continue to be updated [4–6].

Long-term, complicated antiretroviral regimens select very complex viruses, including multi-drug resistant mutations that are currently arcane [7]. Novel mutations may actively participate in NNRTI resistance, and the development of NNRTI resistance may be more complex than previously thought (≥3 NNRTI resistance mutations) [2]. Notably, multiple mutations do not accumulate randomly but appear to be orderly. Evolutionary pathways and the patterns of multiple mutations have been previously selected from a range of quasi-species by drug-selective pressure [8, 9]. Primary NNRTI resistance mutations are also well characterized [10, 11], while, other secondary mutations usually occur in combination with primary NNRTI resistance mutations; whether such mutations further diminish drug susceptibility remains unclear [12].

Various subtypes of HIV lead to features of antiretroviral therapy (ART) in China [13]. We applied a medical model that unifies medicine and management of patients by township health and the Centers for Disease Control and Prevention (CDC). This endows HIV-1 drug resistance strains with features distinctive to our country. Results of HIV-1 drug resistance surveillance in patients who fail first-line antiretroviral therapy showed H221Y usually accompanied the position mutations of T215Y, K103N, Y181 of RT [14]. H221Y is reported to be strongly associated with drug therapy [15], but its role is not clear. Herein, we followed two patients so as to observe the order of appearance of resistance associated mutations, and detected phenotypic sensitivity of recombinant virus that assumed related mutations. Finally, we analyzed the role of H221Y in resistance to commonly used drugs in China (Efavirenz EFV, Zidovudine AZT, Lamivudine 3TC, and Stavudine d4T) and the interactions between H221Y and Y181C.

## Results

### CD4+ T-cells and viral load

**Fig. 1:**
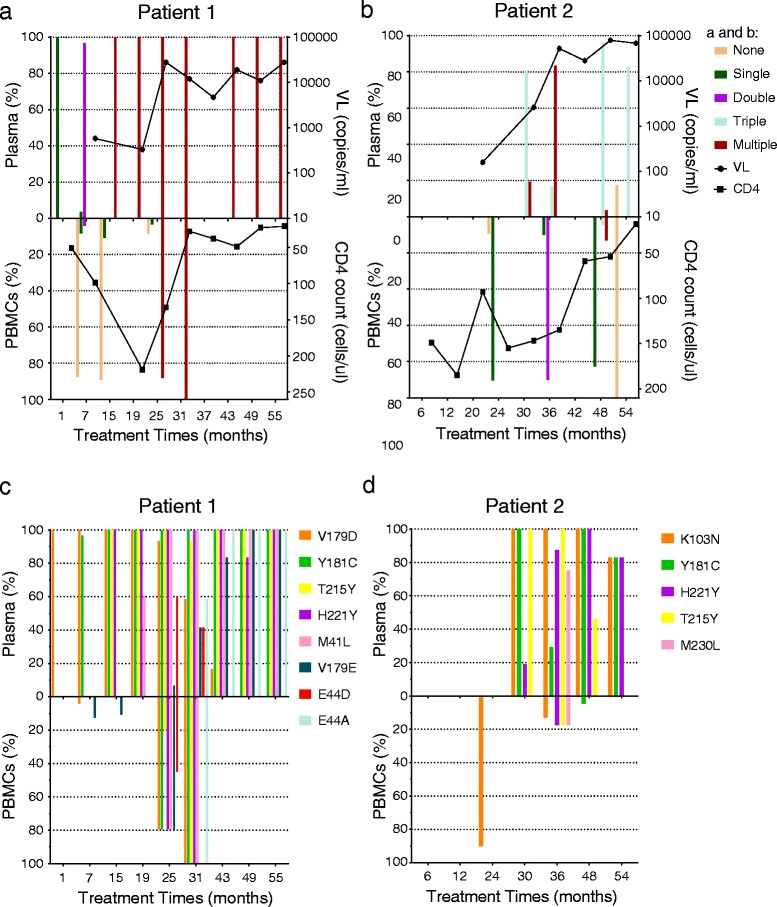
Viral Load (CD4 T cells) evolution, the different time-point, and the proportion of clones with none,single, double,…mutations in plasma (in PBMCs) for the two patients. Viral load was below the sensitivity of Nuclisens® EasyQ kit in the 1^st^ month for patient 1, so we did not show the point in **a**. Viral load was below the sensitivity of Nuclisens® EasyQ kit in the 6^th^ and 12^th^ month for patient 2 and the two points were not show the point in **b. c**, **d** showed the percentage of mutations in RT at each follow-up. Left, y-axis represents mutations in plasma and right, y-axis represents mutations in PBMCs at the same time. Right, x-axis represents follow-up times

For patient 1, CD4+ T-cells increased after initial therapy, especially in the 19^th^ month, where the CD4+ T-cell count was the highest (219 cells/μl). Subsequently, CD4+ T-cell number declined rapidly and dropped to lower than 50 cells/μl in the 31^st^ month. The low CD4+ T-cell numbers continued to the end of the follow-up investigation in the 55^th^ month. However, HIV-1 viral load was maintained at a high level (>10000 copies/ml) since the 25^th^ month, at the same time that CD4+ T-cells began to decline (Fig. 1a). Viral load was below the sensitivity of Nuclisens® EasyQ kit in the 1^st^ month, so we did not show it in the figure. For patient 2, CD4+ T-cells remained at 150 cells/ μl until 30 months of treatment, and then began to decline, dropping below 50 cells/ μl. Viral load was greater than 10^4^ copies/ml from the 36^th^ month on, when CD4+ T-cells began to decline rapidly (Fig. 1b). Viral load was below the sensitivity of Nuclisens® EasyQ kit in the 6^th^ and 12^th^ month.

### Mutation patterns

A clonal sequencing approach was adopted in this study in order to trace the pathway for mutations. We obtained 372 clonal sequences in plasma, with 265 sequences from patient 1 plasma, other sequences from patient 2 plasma. Clonal sequences in PBMC were 197, with 111 sequences from patient 1 plasma, other sequences from patient 2 plasma. These sequences contained different combinations of NRTI and NNRTI resistance mutations.

For patient 1 (Fig. 1c), T215Y was the earliest NRTI mutation to emerge in plasma in the 15^th^ month of therapy, followed by M41L and then by E44D. However, E44A replaced E44D as the last pattern of mutation with respect to M41L/T215Y. In PBMCs, it was M41L/E44D/T215Y that was first detected in the 25^th^ month; with E44A replacing E44D completely by the following visit, and M41L/E44A/T215Y was the last NRTI mutation pattern to be observed. Two mutation patterns, both V179D and V179E, were observed in plasma and PBMCs. V179D was found in plasma from the first month; however, V179E began to replace V179D in the 25^th^ month and completely replaced V179D in the 49^th^ month. V179D first emerged in PBMCs in the 7th month, with only 12.5 %. V179E emerged in the 25^th^ month and completely replaced V179D in the 31^st^ month. In plasma, Y181C emerged in the 7^th^ month, followed by H221Y with 100 % mutation in the 15^th^ month. In PBMCs, Y181C combined with H221Y to emerge in the 25^th^ month and this then remained stable. However, resistance in PBMCs occurred later than in plasma.

For patient 2 (Fig. 1d), we did not gain an adequate quality of sequences in PBMCs, but acquired clonal sequences with high viral load in the plasma sample. In plasma from patient 2, numbers of mutations were very low. T215Y, K103N, and Y181C were detected in plasma with 100 % observed by the 30^th^ month. H221Y emerged with a low mutation percentage (19.20 %). And by the following visit, virus K103N/Y181C/H221Y was associated the same percentage.

### Phenotypic assays of drug susceptibility

Patients in this study were exposed to both NRTI and NNRTI (AZT plus ddI plus NVP). The last stable mutation pattern observed in both patients’ plasma and in PBMCs was the double-mutation Y181C/H221Y, along with others (Fig. 1c, d). We detected the phenotype of the last mutation patterns by recombining patient-derived HIV fragments into a pNL4-3 plasmid. Herein, we then examined the impact of mutations on AZT anti-retroviral therapy. EFV, 3TC, and d4T were not used in the two patients, but are commonly used in HAART in China.

We constructed a recombinant virus by site-directed mutagenesis to analyze impacts of drug susceptibility. We obtained eight recombinant viruses: T215Y/V179E/H221Y/Y181C, T215Y/V179E/Y181C, T215Y/V179E/H221Y, T215Y/V179E, T215Y/K103N/H221Y/Y181C, T215Y/K103N/Y181C, T215Y/K103N/H221Y, and T215Y/K103N. Table 1 shows the mean ± SD of IC50s from three independent experiments and the fold-change in IC50 (FC, calculated by dividing the IC50 for each mutant virus by the IC50 for the wild-type).

**Table 1:** IC50s for 5 viruses and comparison of FC and pNL4-3_*wt*_

Virus		EC_50_^a^ ± SD (nM) (FC^b^)			
NNRTIs	NRTIs	AZT	EFV	3TC	d4T
WT_pNL4-3_		24.80 ± 12.83	14.90 ± 0.35	25.46 ± 3.90	197.87 ± 7.93
V179E/Y181C/H221Y	T215Y	273.43 ± 108.18(11.02)	83.04 ± 25.00 (5.57)**	427.03 ± 225.85 (16.77)	880.20 ± 299.01 (4.04)
V179E/Y181C	T215Y	382.50 ± 216.51 (15.42)^c^	62.94 ± 3.58 (4.23)**	622.10 ± 82.10 (24.43)**	1048.73 ± 229.18 (5.30)^c^
V179E/H221Y	T215Y	1950.67 ± 688.66 (78.65)**	52.48 ± 3.44 (3.52) ^c^	1304.30 ± 304.75 (51.22)**	2170.00 ± 494.11 (10.97)**
V179E	T215Y	501.00 ± 95.98 (20.20)**	34.41 ± 11.97 (2.31)	240.77 ± 34.68 (9.46)^c^	1031.33 ± 350.34 (5.21)^c^
K103N/Y181C/H221Y	T215Y	1337.67 ± 89.49 (53.94)**	503.47 ± 207.80 (33.79)**	807.70 ± 123.88 (31.72)**	7462.67 ± 900.25 (37.72)**
K103N/Y181C	T215Y	52.78 ± 6.79 (2.13)	122.07 ± 12.24 (8.19)**	139.67 ± 41.81 (5.49)	420.60 ± 125.31 (2.13)
K103N/H221Y	T215Y	1393.00 ± 100.80 (56.17)**	154.63 ± 30.80 (10.38)**	264.73 ± 42.13 (10.40)**	422.27 ± 46.19 (2.13)
K103N	T215Y	88.00 ± 12.48 (3.55)	392.70 ± 74.88 (26.36)**	141.60 ± 62.63 (5.56)	471.93 ± 66.35 (2.39)

^a^the 50 % inhibitory concentration

^b^calculated by dividing the IC50 of each mutant virus by the IC50 for the wild-type. Mean ± SD of three independent experiments

^c^IC50 was significantly changed compared with pNL4-3_*wt*_

*P* < 0.05; “**” denotes *P* < 0.01

As shown in Table 1, virus*T215Y/V179E/Y181C/H221Y*, the recombined patient-derived HIV-1 RT fragment, did not exhibit a significant increase with respect to AZT resistance, but did exhibit higher levels of resistance to EFV (up to 5.57-fold). However, it did not enhance resistance greatly to the other two NRTIs tested. T215Y/V179E-containing virus resulted in a dramatic 20.20-fold increase in AZT resistance (*p* < 0.01), and a second increase in 3TC and d4T (*P* < 0.05). Virus with a triple-mutation of T215Y/V179E/Y181C manifested an observed increase in AZT, 3TC, and d4T resistance (*p* < 0.05). In addition, this virus also resulted in a dramatic increase in EFV resistance (*p* < 0.01). With respect to EFV and 3TC effects, patients were not to take medicine in follow-up investigations. The IC50 of different drugs for another triple-mutation, T215Y/V179E/H221Y, presented an effect similar to that with T215Y/V179E/Y181C, but at a different level of significance. Resistance was highly significantly increased with respect to AZT, 3TC, and d4T (*p* < 0.01), while regarding EFV, this increase was significant at the *p* < 0.05 level.

Virus*T215Y/K103N/Y181C/H221Y*, the HIV-1 RT fragment from patient 2, exhibited dramatic increases in IC50 with respect to all the four agents (AZT, 53.94-fold; EFV, 33.79-fold; 3TC, 31.72-fold; d4T, 37.72-fold; *p* < 0.01). However, when H221Y was reversed (T215Y/K103N/Y181C), only the IC50 of EFV was observed to significantly increase to 122.07 ± 12.24 nM (8.19-fold, *p* < 0.01), although this was lower than the 503.47 ± 207.80 nM (33.79-fold). T215Y/K103N resulted in a 26.36-fold increase in EFV (*p* < 0.01). When we added H221Y, the T215Y/K103N virus significantly increased its AZT and 3TC resistance.

### Contributions to drug resistance of mutations

First, we analyzed the resistance contribution of the background mutations. *P* ≤ 0.01 was considered to be statistically significant. The resistance contribution of background mutations was not statistically significant with respect to AZT, 3TC or d4T (F = 1.79, *P* = 0.1968, F = 7.19, *P* = 0.0148 and F = 4.99, *P* = 0.0376) (Table 2). However, the IC50 for virus incorporating Y181C was higher than the others without Y181C with respect to AZT and 3TC (*P* < 0.0001 and *P* = 0.0010); while Y181C did not increase the IC50 of d4T (F = 4.99, *P* = 0.0719). H221Y did not affect the IC50 of three NRTIs (AZT, 3TC, and d4T). The interaction between the two sets was not statistically significant for AZT, 3TC, and d4T.

**Table 2:** Results of statistical analysis of resistance contributed by different sets

Name of drug	Background mutations factor^a^		P (Single seat^b^)		P (Interaction(181*221)^c^)	
	F value	P	181	221	T215Y/V179E	T215Y/K103N
EFV	93.10	<0.0001	0.0002 (F = 43.20)^d^	0.0598 (F = 4.80)^d^	0.3052 (F = 1.20)^d^	0.0003 (38.12)^e^
			0.7404 (F = 0.12)^e^	0.0736 (F = 4.24)^e^		
AZT	1.79	0.1968	<.0001 (F = 25.84)	0.018 (F = 6.69)	0.2270 (F = 1.56)^f^	
3TC	7.19	0.0148	0.0010 (F = 14.94)	0.5356 (F = 0.40)	0.2513 (F = 1.40)	
d4T	4.99	0.0376	0.0719 (F = 4.99)	0.4400 (F = 0.62)	0.2269 (F = 1.56)	

^a^Analyze if it is statistical significance between the impact of T215Y/V179E and T215Y/K103N on the IC50 of different drugs. Here, T215Y/V179E and T215Y/K103N are the background mutations factors

^b^Analyze the impact of single mutations (Y181C or H221Y) on the IC50 of different drugs

^c^Analysis of the interaction between 181 and 221 on the different background mutations factors. Due to the difference is significant of impact of T215Y/V179E and T215Y/K103N on EFV (F = 93.10, *P* < 0.0001)

^d^and ^e^analyze the interaction between 181 and 221 on the background of T215Y/V179E and T215Y/K103N respectively. f is the P of the interaction between 181 and 221 ignoring the background mutations on different drugs

*P* ≤ 0.01 was considered to be statistically significant

For EFV, the IC50 for virus with the T215Y/V179E mutation was higher than with T215Y/K103N, suggesting that the background factor affected the IC50 significantly (F = 93.10, *P* < 0.0001). Regarding T215Y/V179E as the background, Y181C expressed significant increases in EFV resistance, but H221Y did not. The interaction between 181 and 221 in EFV was also not statistically significant (F = 1.20, *P* = 0.3052). In a T215Y/K103N background, neither H221Y nor Y181C expressed significant changes in EFV resistance (F = 0.12, *P* = 0.7404 and F = 4.24, *P* = 0.0736), but the interaction between 181 and 221 was statistically significant (F = 38.12, *P* = 0.0003).

## Discussion

We observed the dynamic changes in CD4+ T-cell number and viral load from the start of therapy. During the first 20 months approximately, therapy may be effective, with corresponding changes in CD4+ count and viral load. However, therapy may fail from the 20^th^ month on. Viral load increased markedly and CD4+ T-cell count dropped rapidly in our study. One possible explanation for the effectiveness of HAART is that mutations that confer resistance to one drug class can increase the susceptibility of viruses to other classes [16, 17]. However, resistance mutations did not emerge individually, but rather in a combined fashion.

We detected the RT genotype of the virus in plasma and PBMCs at every follow-up visit, and we analyzed the mutations that emerged in plasma and PBMCs using clonal sequencing. No mutations were shown during 43–55 months in patient 1 because we did not acquire a sufficient number of sequences due to so few PBMCs being present. However, related mutations emerged in PBMCs later than in plasma as in a previous study [18]. Herein, T215Y and V179D were observed in plasma first compared to PBMC, as with Y181C. However, different pathways were displayed in plasma and PBMCs. In plasma, emergence of H221Y followed Y181C for about one half–year, but was combined with Y181C in PBMCs. Overall, we observed a change in not only the number of mutations over time (from less to more, then to less), but also in the specific combination of mutations that evolved (to achieve stable for about 48 months). At the end of the current investigation, we found that the mutation at 179 exited stably with V179E, but not initially with V179D. It is reported that a differential genetic barrier was found for V106M, V108I, P225H in different HIV-1 subtypes for NNRTI resistance-related substitutions [19]. It may be the reason for the phenomenon of V179D/E. Resistance mutations usually are polymorphic, and include at least two nucleotides. The changing of amino acid spatial structure affect the affinity of drugs and target to cause resistance. This may be another reason for more stable of V179E than V179D. However, the last patterns of correlated mutations may be the result of pharmacologic pressure imposed by the drug regimens [2, 7]. This suggests that other treatment regimens may lead to the development of pathways that are partially different from those that we observed. In 1994, Larder summarized a series of mutations associated with T215Y and the sequential appearance of these changes [20]. This report indicated that K70R was followed by T215Y and accumulated the cluster mutation M41L/D67N/K70R/T215Y (K219Q), but herein we observed no cluster. We rather considered H221Y as a novel mutation along with Y181C.

More complex combinations of RT mutations confer more complex co-resistance. A study detected that multiple mutations (K103N/Y181C/H221Y, K103N/Y181C, K103N/H221Y) significantly increased NVP resistance [21]. Herein, we determined the impact of associated mutations with respect to AZT medications taken by patients, d4T (with the same resistance mechanisms as AZT), 3TC (whose resistance mechanisms were increased with reference to RT discrimination properties [22]), and EFV. Classical key mutations (TAMs, M184V, K103N and so on) recruited in HIV drug resistance database are responsible for either drug-specific resistance or cross resistance; and along with these, viral strains demonstrate non-canonical changes in treated patients whose contribution to the phenotype is unknown [20]. T215Y, a classical mutation, is suggested to cause AZT and d4T resistance; and V179D/E is considered to be an NNRTI mutation, by itself reducing NVP and EFV susceptibility approximately 2-fold [23]. In the present study, we detected the contribution to AZT, 3TC, d4T and EFV resistance by the double mutation T215Y/V179E. Our results showed that the co-presence of T215Y/V179E significantly increased AZT and d4T resistance respectively. However, we could not sure if the effect of single V179E is important and further research about role of V179E is needed. However, V179E is associated with low-level resistance to EFV in HIV drug resistance database [23]. But resistance to EFV of T215Y/V179E is not significant in our study. This may be affected by T215Y. It is not reported that T215Y is associated with resistance of 3TC [23]. We found resistance of T215Y/V179E to 3TC was significant and this may be affected by V179E.

H221Y is a nonpolymorphic accessory NNRTI-selected mutation that usually occurs in combination with Y181C [24]. It is frequently selected in patients receiving rilpivrine (RPV) [25]. Alone H221Y has minimal detectable effects on NNRTI susceptibility [26]. According to the mutation patterns that emerged in plasma, i.e., Y181C followed by H221Y. We separately compared the resistance of T215Y/V179E/Y181C and T215Y/V179E/Y181C/H221Y to the wild-type virus. The triple-mutation T215Y/V179E/Y181C led to a dramatic increase in resistance in the wild-type virus to all four drugs evaluated. However, T215Y/V179E/Y181C/H221Y only resulted in a marked increase in EFV. H221Y slightly decreased T215Y/V179E/Y181C resistance to AZT, d4T and 3TC. With respect to EFV, Y181C, and H221Y both showed increased resistance. However, these sets of resistance mutations (Y181C/H221Y) conferred cross-resistance to all three NRTIs. Y181C is a nonpolymorphic mutation selected in vitro by NNRTIs (NVP, EFV, RPV and ETR) and it is not reported relating to NRTIs. We found Y181C attenuated resistance of T215Y/V179E to AZT, but it was not significant. Additionally, Y181C did not modify resistance of T215Y/V179E to d4T and led to an increase in the mean IC50 value from 240.77 ± 34.68 nM for T215Y/V179E to 622.10 ± 82.10 nM for the triple-mutation with respect to 3TC.

T215Y is interpreted to be resistant to AZT and d4T; and its co-presence with K103N, a mutation to the first-generation NNRTI, did not increase the IC50 with respect to AZT and d4T. In other words, K103N may compensate resistance as a result of T215Y with respect to AZT and d4T. Adding Y181C to T215Y/K103N lowered the IC50 of AZT to insignificance. Y181C and H221Y also decreased the impact of T215Y/K103N on EFV, and their combination with T215Y/K103N augmented the IC50 of EFV. To confirm the role of Y181C, H221Y and any interaction between them, we analyzed the results of IC50s using appropriate statistical methods. We first analyzed the impact of background mutations on the various agents. Results showed that there was no difference in the IC50s of AZT, 3TC, or d4T between T215Y/V179E and T215Y/K103N. It is likely that effect of T215Y predominates as regards the resistance to AZT, 3TC, and d4T. The IC50 of virus incorporating T215Y/K103N was higher than the virus with T215Y/V179E (*P* < 0.0001). One possible explanation for this is that the impact of K103N on EFV resistance may be greater than that of V179E. We therefore analyzed the contribution of the single mutations Y181C and H221Y. Y181C was demonstrated to decrease the IC50s of AZT and 3TC, and H221Y increased the IC50 of AZT. Y181I/C, which constituted NVP-selected mutations, was reported not only to confer cross-resistance to d4T [27, 28], but also to improve sensitivity to AZT [20]. Herein, we did not obtain an effect of Y181C with respect to d4T. However, interaction between the two sites was not significant. For EFV, only Y181C increased the IC50 in a T215Y/V179E background. Contribution to resistance for H221Y was insignificant compared with Y181C. Dramatically, Y181C and H221Y expressed synergism in a T215Y/K103N background of. Patients should adjust therapy regimen as quickly as possible for better result once T215Y/K103N/Y181C/H221Y emerging. Since contributions to resistance for multiple mutations were not geometrically additive, it is conceivable that a greater number of mutations or associated mutations than are currently known are involved in the development of drug resistance [7]. How these mutations cause resistance and alter replicative capacity is unclear. Future studies will be necessary to better elucidate and highlight their mechanisms of action both in *vitro* and in *vivo*. Along with more complex patterns, it is more different to mastery the mechanism of resistance and replication. Although, viral evolution pathways toward drug resistance may proceed through distinct steps and at different rates among different HIV-1 subtypes [8]. To study the interaction between different mutations is contribute to partly understand the pathways of mutation patterns under different drugs pressure. However, similar evaluation in more subtypes B’ and other non-B’ subtypes phenotypic test of HIV-1 is necessary, because of data in this study based on a recombine of subtype B’ and pNL4-3 wild-type. Furthermore, not only alterations in RT function of a mutation may contribute to resistance and replication, but interaction between mutations give some information on the impact of these mutations on virus replication and drug resistance.

## Conclusions

Data in this study suggests that pathways of viral evolution toward drug resistance appear to proceed through distinct steps and at different rates among HIV-1 infectors. Phenotypic resistance using recombinant virus strains with different combination of mutation patterns reveals that interactions among mutations may provide information on the impact of these mutations on drug resistance. All the result provides reference to optimize clinical treatment schedule.

## Material and methods

### Patients

Two patients from a HIV-1 drug resistance surveillance cohort in Henan province were selected after informed consent. Data revealed that they are of Han ethnicity and not habituated to alcohol. The two male patients were infected with HIV-1 by commercial plasma collection and their infections were validated in July of 2003. They were given regimens of NRTIs plus NNRTIs [AZT plus Didanosine (ddI) plus Nevirapine (NVP)] in September, and we have followed them for ten time-periods over 55 months, with intervals of five to six months each. We collected 10 ml samples of anti-coagulated whole blood each time, centrifuged the samples to obtain plasma and peripheral blood mononuclear cells (PBMCs), and stored them at -80 °C. Patient 1 (35 years old) complied with treatment regimens well, while patient 2 (45 years old) discontinued the drugs during the follow-up periods.

### CD4+ T cell and plasma HIV-1 viral load

A flow cytometer (FACSCalibur, BD, America) was used to count CD4+ T cells according to the manufacturer’s instructions, using BD TriTESTT[M] CD3FITC/CD4PE/CD45PerCP for fluorescence labeling. Plasma HIV-1 viral loads were measured by Nucleic Acid Sequence Based Assay (NASBA) according to Nuclisens® EasyQ kit (BioMerieux, France) instructions.

### Clonal sequencing of HIV-1 in plasma and PBMCs

Clonal sequencing was performed according to reference [29]. RNA and DNA were extracted from plasma and PBMCs as templates for nested PCRs, respectively. RNA and DNA were amplified using outer primers PLA-1/PLA-2 and inner primers PLA-3/PLA-4 [14, 17]. PCR products (2.1 kb, HXB2: 2147-4308 bp) were independently cloned into pMD-18 T, and a single clone was sequenced. Each sequence reflected the genotype of an independent viral genome. To track the mutation patterns, we analyzed sequences from each follow-up time.

### Recombinant virus construction and viral stocks preparation

Recombinant virus was constructed as previously described [21]. Site-directed mutagenesis was carried out to reverse H221Y and/or Y181C of pMD-18 T, which harbored T215Y/V179E/Y181C/H221Y and T215Y/K103N/Y181C/H221Y, respectively. A 643 bp HIV-1 RT fragment (HXB2: 2843 nt-3485 nt) was obtained by Age I (A/CCGGT) and Sbf I (CCTGCA/GG), and this replaced the corresponding sequence in pNL4-3 *pol*. The following mutations were introduced into RT of recombinant pNL4-3 plasmids: T215Y/V179E/Y181C/H221Y, T215Y/V179E/Y181C, T215Y/V179E/H221Y, T215Y/V179E, T215Y/K103N/Y181C/H221Y, T215Y/K103N/Y181C, T215Y/K103N/H221Y, and T215Y/K103N. Recombinant pNL4-3 wild-type (pNL4-3_wt_) and mutant viruses were generated by the transfection of the corresponding plasmid DNAs into HEK293T cells using Lipofectamine 2000 according to the manufacturer’s instructions (Invitrogen, America). Transfection supernatants were harvested at 48 h post-transfection and infected MT-2 cells and viral cultures were grown in 4 to 6 days. Supernatants were stored at -80 °C and the presence of the desired mutations was verified by amplification and sequencing.

### Phenotypic drug susceptibility assays

Phenotypic susceptibility analysis of the RT inhibitors (EFV, AZT, 3TC, and d4T) was performed with recombinant viruses in TZM-b1 (JC53) cells as described previously [18]. Briefly, drugs at various concentrations were added to TZM-bl cells (10^4^ cells/well) in 96-well plates and cells were grown in 100 μl of Dulbecco’s minimal essential medium (DMEM; Gibco, America) supplemented with 10 % fetal bovine serum (Gibco, America) and 1 % penicillin-streptomycin [30]. Immediately after drug addition, cells were infected with pNL4-3_wt_ or mutant viruses normalized by a 50 % tissue culture infectious dose (TCID_50_). After virus and cells were co-cultured for 48 h at 37 °C in 5 % CO2 in compressed air, relative luminescence units (RLU)/well were measured using a luminometer (Wallik 1420; Perkin Elmer, America) according to the Bright-GloTM Luciferase assay system (Promega E2650, Promega, America) instructions. The 50 % inhibition concentration (IC_50_) was calculated using GraphPad Prism (GraphPad Software, Inc. America) by plotting inhibition curves of the percentage of inhibition of luciferase activity *versus* log_10_ drug concentration [31, 32].

### Statistical analysis

We used multiple testing statistical methods to assess the IC_50_ differences between pNL4-3_wt_ and mutant viruses and a nonparametric test was used for corrections. We used quantitative data hypothesis testing in a factorial design to analyze the role of H221Y and the interactions between 181 and 221. A false discovery rate of 0.05 was used to determine statistical significance. *P* ≤ 0.01 was considered to show statistical significance among mutational viruses. Analyses were performed using SAS (SAS Institute, Inc., America).

### Ethical considerations

The study was approved by the Ethical Board of the Beijing Institute of Microbiology and Epidemiology. All patients were selected after filing written informed consents. Written informed consents were obtained from the patients for usage of the individual patient details. Data were managed anonymously.

## Abbreviations

(3TC): Lamivudine
(AZT): Zidovudine
(d4T): Stavudine
(EFV): Efavirenz
(HAART): Highly active antiretroviral therapy
(HIV-1): Human immunodeficiency virus type-1
(NNRTIs): Non-nucleoside reverse transcriptase inhibitors
(NRTIs): Nucleoside reverse transcriptase inhibitors
